# A 3D *ex vivo* mandible slice system for longitudinal culturing of transplanted dental pulp progenitor cells

**DOI:** 10.1002/cyto.a.22680

**Published:** 2015-05-11

**Authors:** John S. Colombo, Rachel A. Howard‐Jones, Fraser I. Young, Rachel J. Waddington, Rachel J. Errington, Alastair J. Sloan

**Affiliations:** ^1^ School of Dentistry, University of UtahSalt Lake CityUtah; ^2^ Tissue Engineering and Reparative Dentistry, Cardiff Institute of Tissue Engineering and Repair, School of Dentistry, Cardiff University, Heath ParkCardiff, WalesUnited Kingdom; ^3^ Institute of Cancer and Genetics, School of Medicine, Cardiff University, Heath ParkCardiff, WalesUnited Kingdom; ^4^ Neuroscience and Mental Health Research Institute, School of Medicine, Cardiff University, Hadyn Ellis BuildingCardiff, WalesUnited Kingdom

**Keywords:** mesenchymal stem cell, organ culture, high‐content imaging

## Abstract

Harnessing mesenchymal stem cells for tissue repair underpins regenerative medicine. However, how the 3D tissue matrix maintains such cells in a quiescent state whilst at the same time primed to respond to tissue damage remains relatively unknown. Developing more physiologically relevant 3D models would allow us to better understand the matrix drivers and influence on cell‐lineage differentiation *in situ*. In this study, we have developed an *ex vivo* organotypic rat mandible slice model; a technically defined platform for the culture and characterization of dental pulp progenitor cells expressing GFP driven by the β‐actin promoter (cGFP DPPCs). Using confocal microscopy we have characterized how the native environment influences the progenitor cells transplanted into the dental pulp. Injected cGFP‐DPPCs were highly viable and furthermore differentially proliferated in unique regions of the mandible slice; in the dentine region, cGFP‐DPPCs showed a columnar morphology indicative of expansion and lineage differentiation. Hence, we demonstrated the systematic capacity for establishing a dental pulp cell‐micro‐community, phenotypically modified in the tooth (the “biology”); and at the same time addressed technical challenges enabling the mandible slice to be accessible on platforms for high‐content imaging (the biology in a “multiplex” format). © 2015 The Authors. Published by Wiley Periodicals, Inc.

Mesenchymal stem cells (MSCs), multi‐potent cells from which connective tissues arise, are currently the focus of intense scientific interest from the standpoint of regenerative medicine and tissue engineering. Harnessing the MSC population to initiate repair has wide ranging implications in many tissue types, including teeth, bone, and the cardio‐vascular system 1, 2, 3, 4, 5, 6. In the tooth, endogenous MSCs present in the dental pulp are known to be capable of migration to a site of tissue damage and differentiation into odontoblasts enhancing capacity for the active synthesis of reparative matrix 7, 8, 9. Evidence is emerging which points to mineralized matrix itself as a significant source of relevant growth factors (BMP‐2, VEGF, and TGFβ1 have been shown to have a stimulatory effect of MSCs resident within the dental pulp 10, 11). The mechanistic role of the matrix in which dental pulp progenitor cells (DPPCs) reside is not clearly defined—it is comprised primarily of collagen types (I&III), fibronectin and proteoglycans 12. DPPCs are heterogeneous in nature and numerous studies suggest significant clonal variation may exist. Such heterogeneity may be explained by the possible tissue niches which may exist within the tissue including undifferentiated mesenchymal dental papilla cells, neural crest stem cells 13 and a third niche surrounding the extensive vascular network within the tissue (pericyte/perivascular population). Current isolation methods make no distinction between these different populations and individual clones may not be from the “same” cell source. This has led to studies investigating side populations of cells to enrich for lineage differentiation. Such reports and the requirement to understand the cells in their niche environment make them a suitable source for this study. Since the isolation of multi‐potent DPPCs 9, there has been an increasing interest in inducing these cells to regenerate both soft pulp and mineralized dentine tissues, effectively utilizing the capacity of the tooth for self‐repair and preserving the vitality of the dental pulp 6. If multi‐potent DPPCs can be rapidly recruited to a site of tissue destruction and induced to differentiate into reparative phenotypes, it is conceivable that vascularization, soft tissue integrity and mineralized tissue could all be restored, preserving the vitality and integrity of the dental pulp. The questions arise as to how a complex three dimensional matrix environment is involved in the maintenance of DPPC cells in a quiescent progenitor cell state and at the same time are primed to respond to tissue damage. The critical matrix‐cell interactions that influence and link these processes are not currently understood. As the therapeutic intent becomes aimed at eliciting a repair response from endogenous populations of cells, there is both a technology and intellectual requirement for cellular model systems that enable the systematic interrogation of cell‐dental pulp niche interactions.

The transition from monolayer cell culture on the flat substrate of a plastic vessel (sometimes coated) to a three‐dimensional (3D) environment, matrix or scaffold with a 3D architecture is well established, and in particular for biomedical applications in tissue engineering and stem cell research 14, 15, 16, 17, 18. The prospect of developing more physiologically relevant 3D models systems for use in *in vitro* high‐content screening however, remains open to major improvement. The current‐status is still some way off from providing fully validated and robust 3D culture methodologies and tools—particularly where both the cell‐cell heterogeneity and phenotypic expression for chemo resistance are adequately accommodated. We take the view that with a novel capacity for using an organotypic culture model, there is an opportunity for constructing and understanding better defined micro‐communities. There is a drive toward cell‐based assays that enable functional measurements of complex cellular behavior. On the other hand, there exists a need to make kinetic cell‐based assays 3D, simple to perform and include readouts of the micro‐environment. High content‐screening also requires the process to be relevant yet ensure accuracy, reproducibility, and scalability.

Thus, the aim of the current work was the development of a rat mandible slice (tooth) culture system, which has provided the “organotypic” soft and hard tissue environment to assess the molecular and cellular responses of DPPCs growing and differentiating in this complex biomaterial. In particular, we focus on a 3D model that enables optical access to the functional identification and measurement of the DPPCs; and where the overriding imaging challenge has been to contend with both hard and soft tissues. Our proposed mandible slice model has provided the micro‐environment to receive GFP‐actin expressing DPPCs, enabling us to determine phenotypic expression of biomarkers, cell spread and orientation throughout the matrix as well as their capacity for proliferation within their native tissue environment. Specifically the work addresses the design, optimization and implementation of a human mandible slice suitable for automated high‐content clonogenic/proliferation assays.

## Materials and Methods

### GFP Cell Isolation

DPPCs were isolated from the dental pulp of GFP “green rats” [SDTg (CAG‐EGFP) CZ‐004 Osb] produced by the method of Okabe and colleagues whereby ubiquitous production of green fluorescent protein is driven under control of a chicken β‐actin promoter 19. Healthy animals were bred and maintained according to the NIH guide for care and use of laboratory animals, approved by the University of Reading animal usage ethics committee and were housed in the Biological Resource Unit. Four‐week old male rats were sacrificed in accordance with Schedule 1 of the UK Animal (Scientific Procedures) Act 1986 and dental pulps were extracted from both mandibular and maxillae incisors. Pulpal tissue was digested for 1 hour at 37 °C with a 4 µg/mL collagenase‐dispase solution (Roche) made up in DPPC medium: α‐MEM media containing nucleosides (Life Technologies) and supplemented with 20% (v/v) fetal bovine serum (Life Technologies), 100 µM L‐ascorbic acid (Sigma), and 100U/mL Penicillin, 100μg/mL Streptomycin Sulphate. The resulting single cell suspension was made up to 1 × 10^4^ cells/ml in DPPC medium. Cells were seeded onto fibronectin‐coated plates to enrich for a progenitor cell population based on their increased expression of α_5_β_1_ integrins 20. Plates were coated overnight at 4 °C using a 10μg/mL solution of human plasma fibronectin (Sigma, UK) diluted in 1× PBS+ (1× PBS supplemented with 0.1 mM MgCl_2_ and 0.1 mM CaCl_2_). Fibronectin‐adherent cells were isolated after 20 min by removal of all non‐adherent cells in suspension. Clonal populations were isolated using cloning rings and subsequently expanded for characterization and experimental use (referred to as cGFP‐DPPCs).

### cGFP‐DPPCs Cell Culture and Characterization

cGFP‐DPPC populations were cultured in DPPC medium and maintained at 37 °C, 5% CO_2_. Cells were passaged when they were 80% confluent and re‐seeded at a density of 2 × 10^4^cells/cm^2^. Cell counts were performed at each passage and population doublings calculated. Cells from a single clone, AVII‐d, were used for experiments between passages 10 and 14. RNA was extracted from AVII‐d at passage 12 using the traditional phenol/chloroform method. RT‐PCR was performed for CD73, CD90, CD105, CD45, CD34, CD146 and Osteopontin (Primer sequences; CD90 Fwd 5' CCTGACCCGAGAGAAGAA 3', Rev 5' TGAAGTTGGCTAGAGTAAGGA 3'; CD73 Fwd 5'GGTGTGGAAGGACTGATTG 3', Rev 5' CCGACAGAGAGAACTTTATATGG 3'; CD105 Fwd 5' ACATGGTGCCCACACCCGCAGCTGGCA 3', Rev 5' CACTGCCACCACGGGCTCCCGCTTGCT 3'; CD45 Fwd 5' CTCACCACACTCACGGCTGCTCCCAGCG 3', Rev 5' GCAGGGCCATTTCGTTGCACCCTCCCAA 3'; CD34 Fwd 5' GTCACACTGCCTACTACTTC 3', Rev 5' TCCTCGGATTCCTGAACAT 3' and Osteopontin Fwd 5' TCCAAGGAGTATAAGCAGAGGGCCA 3', Rev 5' CTCTTAGGGTCTAGGACTAGCTTGT 3'). PCR products were sequenced by Central Biotechnology Services, Cardiff using an Applied Biosystems 3130xl 16 capillary Genetic Analyzer.

### Flow Cytometry and Simple DPPC Profiling

To assess the proportion of GFP expressing DPPCs within the population of isolated DPPCs, 80% confluent cells were detached using accutase (Sigma) and run on a FACSCalibur (BD biosciences) flow cytometer, with 10,000 total events being collected. Simple gating FSC vs. SSC allowed us to identify the viable fraction. From there the profile of the GFP expression was elucidated.

### A Six Step Method to Establish the Mandible Slice Organ Culture Model

**Figure 1:**
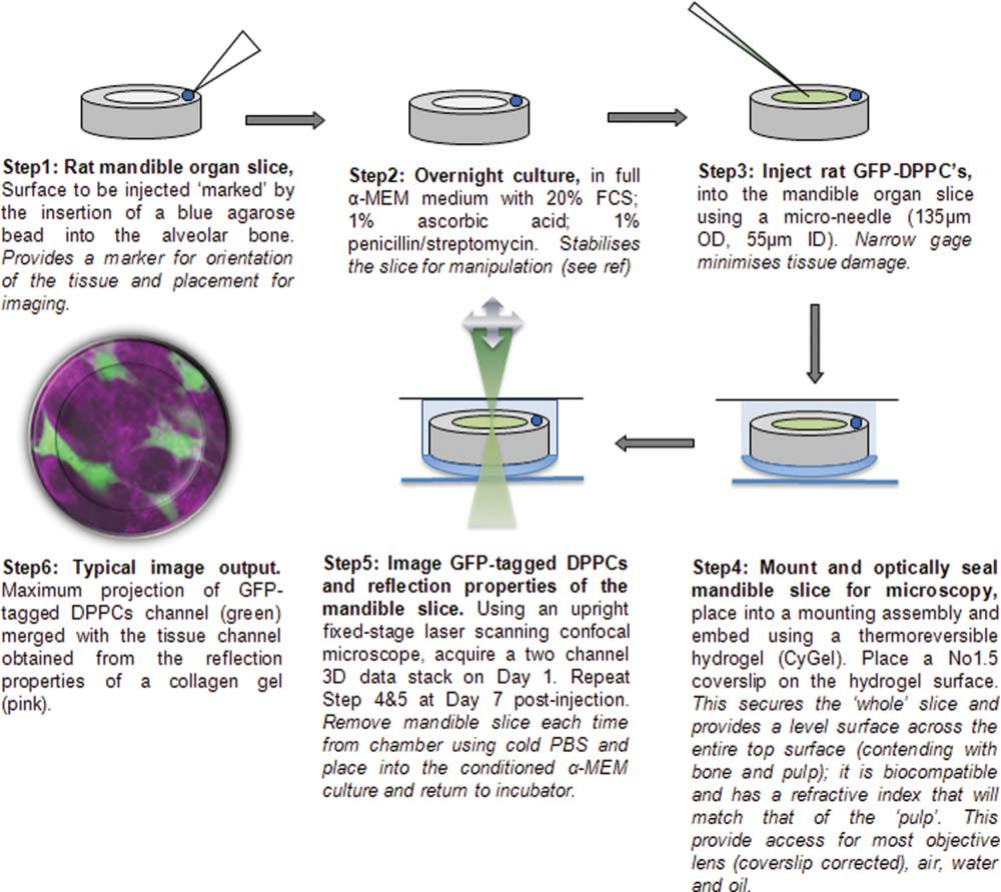
“Organotypic model” to identify transplanted cells growing in their native tissue. Experimental methodology, showing the step‐by‐step process of cell injection (expressing constitutively‐active GFP) into rat mandible slices marked with an agarose bead (fiducial mark, to ensure tissue orientation), culture and subsequent 3D imaging at days 1 and 7 post‐injection. [Color figure can be viewed in the online issue, which is available at wileyonlinelibrary.com.]

The overall approach was based on our established mandible organ slice cultures 18 and adapted for time series laser scanning microscopy (Fig. 1 and Supporting Information Fig. S1).

#### Step 1. Retrieving the mandible organ slice

Animals were sacrificed under schedule 1 of the UK Animals Scientific Procedures Act, 1986 by a qualified technician at the Joint Biological Services Unit, Cardiff University for harvesting of tissue. Briefly, mandibles were removed from euthanized 28‐day old Wistar rats and cut into 2 mm sections using an isomet bone saw (Buehler). A single blue agarose bead (BD Biosciences) was placed in the alveolar bone on the injected side of each mandible slice, in order to aid orientation of the tissue for imaging at each time point

#### Step 2. Stabilizing the mandible slice in culture

These slices were then stabilized in culture for 24 hours in full‐DPPC medium prior to injection with the cGFP‐DPPCs.

#### Step 3. Injection of cGFP DPPCs into mandibles

80% confluent cGFP‐DPPCs were detached using accutase (Sigma), centrifuged, counted using a hemocytometer and resuspended in culture media at a concentration of 2 × 10^6^ cells/mL. Cells were loaded into a nanofil syringe (World Precision Instruments) with a 35 g microneedle. 1 μL, containing 2,000 cGFP DPPCs was injected into the pulp region of each mandible slice. Mandible slices were cultured for up to 7 days at 37 °C, 5% CO_2_. Sham injected mandibles were injected with 1 μL of PBS (Supporting Information Fig. S2).

#### Step 4. Preparation of injected mandible slices for microscopy

In order to maintain an enclosed and level platform for imaging cell injected mandible slices, chambers were made from inverted 1.5 mL Eppendorf tube lids and attached to plastic petri dishes using superglue. Within the chambers provided by the inverted tube lids, mandible slices were suspended in CyGel™ (Biostatus, Shepshed, UK). CyGel is a thermo‐reversible hydrogel; it is a viscous liquid at room temperature, but a gel at 37 °C. Furthermore, it is compatible with live cells and biomaterials and thus acts as a suitable support and immobilizing matrix for the mandible slice. Optically it provides the crucial interface between the biomaterial and No 1.5 coverslip, leveling the surface and contending with the diversity of the surface from bone (a rough surface) to the pulp (a more elastic material). After suspension within the imaging chambers and placement of the coverslips, mandible slices were placed in an incubator at 37 °C for 3 min, allowing the CyGel to solidify around them. After imaging, slices were removed from the imaging chambers by gentle rinsing in sterile 4 °C PBS, cooling and liquefying the CyGel, allowing retrieval. Subsequently, slices were returned to culture in 24‐well plates containing DPPC medium.

#### Step 5. Confocal laser scanning microscopy (CLSM), standard set‐up, and acquisition

Mounted slices were imaged using an upright confocal microscope (Nikon Eclipse FN1 Fixed‐stage with a Radiance 2100 scan head, Zeiss, UK). Dual channel images were collected simultaneously (GFP fluorescence 488 nm excitation and 530/30 emission; and reflection at 488 nm of the cell‐matrix tissue (a polarizer was used to remove mottle)). A simple LUT was used to ensure that there was no saturation of signal at both ends of the grey scale levels (ie no pixels at 0 and 255). The x,y position of the data collection was always made with reference to the agarose bead. Z stack images were collected at 2 µm increments with a Nikon Plan Fluor x10, 0.3 NA lens; importantly, the reflection image was used to ensure the collection regimen was always the same, the two‐surface reflection of the coverslip acted as a reference point which ensured that the data was collected from inside the mandible slice toward the coverslip, this enabled the location of similar depth comparison between DAY 1 and DAY 7. Mandible slices were imaged at both 1 and 7 days after injection with cGFP‐DPPCs (*n* = 3). Correction of z‐focus distortion was adopted to calculate actual depth of each z‐region, the correction factor in this case was a ratio of the refractive index air:CyGel (i.e., 1.365) 21

#### Step 6. Projection and image analysis

Images were visualised using Metamorph imaging software. Maximum projection images of the pulp surface, pulp sub‐surface and dentine region of the injected tooth slice were created for DAY 1 and DAY 7 post injection using equal Z stack images. Equivalent z‐depth maximum projection images of each region were made to perform further simple image analysis, to determine features of cell growth and location (patterning) over the two time points. Total area of GFP fluorescence (in pixels) was calculated using Metamorph region measurements, to provide a global index of cell density or spread for a given region of the mandible slice. Morphology filters were used to extract features from cell ‘blobs’ and provided the means for the estimate of number and centroid for each cell, again in a designated region. Each of the cell ‘blobs’ represented a total pixel area ranging from 55 to 120; after applying a median filter to each image, a Top Hat filter (shape circle (three pixels diameter), was used to detect the bright features across the image and this corresponded to the location of individual cells 22

## Results

### Characterization of cGFP‐DPPCs

**Figure 2:**
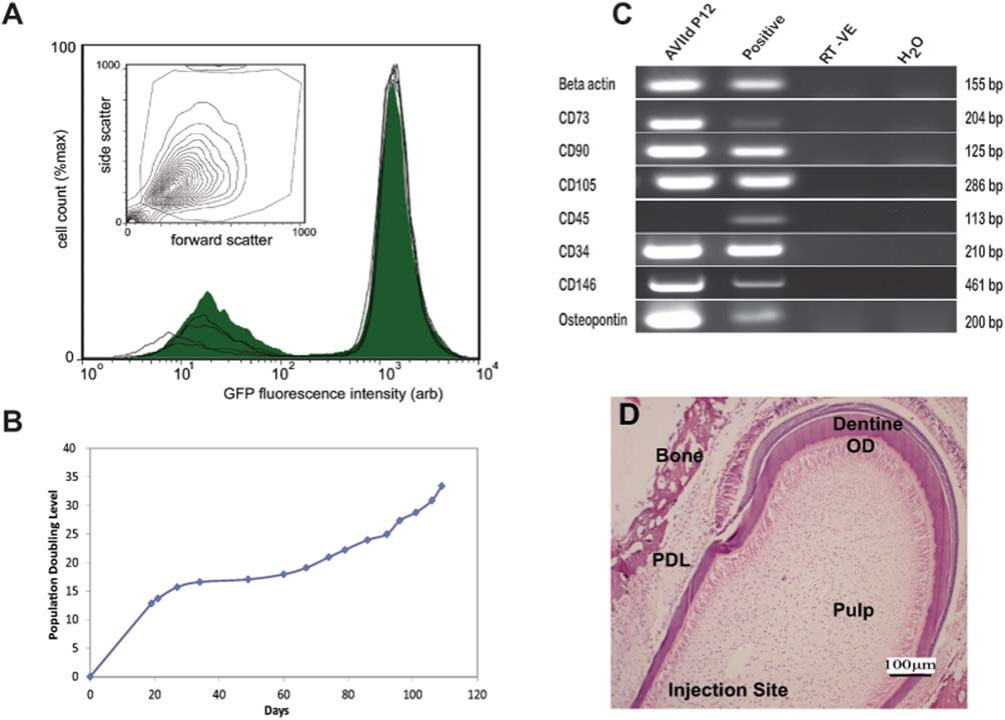
cGFP‐DPPC characterisation. **A**: The stability of GFP expression profiles were obtained for cell populations grown over 3 days on tissue culture plastic. Flow cytometry histogram to demonstrate the extent and levels of GFP‐expression per cell (Graph insert FSC/SSC gating of live cells to remove debris). Average GFP expression 80.66% ± 7.1 and geometric mean of GFP fluorescence 1463 ± 42.2 (arb units)). **B**: Time‐dependent population doubling levels for cGFP‐DPPCs (note cells are still expanding when used at passage 12). **C**: PCR analysis indicates positive expression of known mesenchymal markers; CD73, CD90, CD105, pericyte marker CD146 as well as osteopontin and CD34 and negative expression for CD45. **D**: Hematoxylin and eosin stained mandible slice cultured for 7 days shows different regions of a mandible slice (note the orientation is similar to images shown in Fig. 3). It also demonstrates that normal tissue architecture (pulp, dentine, odeontoblasts (OD), periodontal ligament (PDL) and bone) is maintained after injection and culture. [Color figure can be viewed in the online issue, which is available at wileyonlinelibrary.com.]

Population analysis using flow cytometry showed a high level of GFP‐expression (FL1‐H geometric mean 1463 ± 42.2 (arb units)) which averaged 80.66% ± 7.1 for the cGFP‐DPPCs (Fig. 2A). This stable high level of GFP‐expression in the positive fraction is important for identification of the injected fraction into the mandible tissue. Since β‐actin is a housekeeping gene in the DPPC lineage (see Fig. 2A), GFP expression controlled under its promoter ensures high protein levels. This is particularly important when imaging these live cells transplanted into the highly scattering environment of the pulp to ensure optimal detection. cGFP‐DPPCs demonstrated a sustained population doubling after an extended time in culture and were still dividing at the passage number at which they were used experimentally (passage 10‐14) (Fig. 2B). cGFP‐DPPCs were found to positively express mesenchymal stem cell markers CD73, CD90 and CD105, and were negative for the hematopoietic marker CD45 consistent with standard MSC characterisation 23, 24. In addition to the mesenchymal stem cell markers, these cells express CD146, indicative of pericytes but have additional expression of CD34, suggesting potential presence of transit amplifying progenitor cells 25. These cells also demonstrated positive expression of osteopontin, an early mineralized tissue marker associated with early attachment, at the mRNA level (Fig. 2C). The tooth slices demonstrated maintenance of the tissue architecture following injection of the cGFP‐DPPCs (Fig. 2D).

### Injection of cGFP‐DPPCs Into Mandible Slices

**Figure 3:**
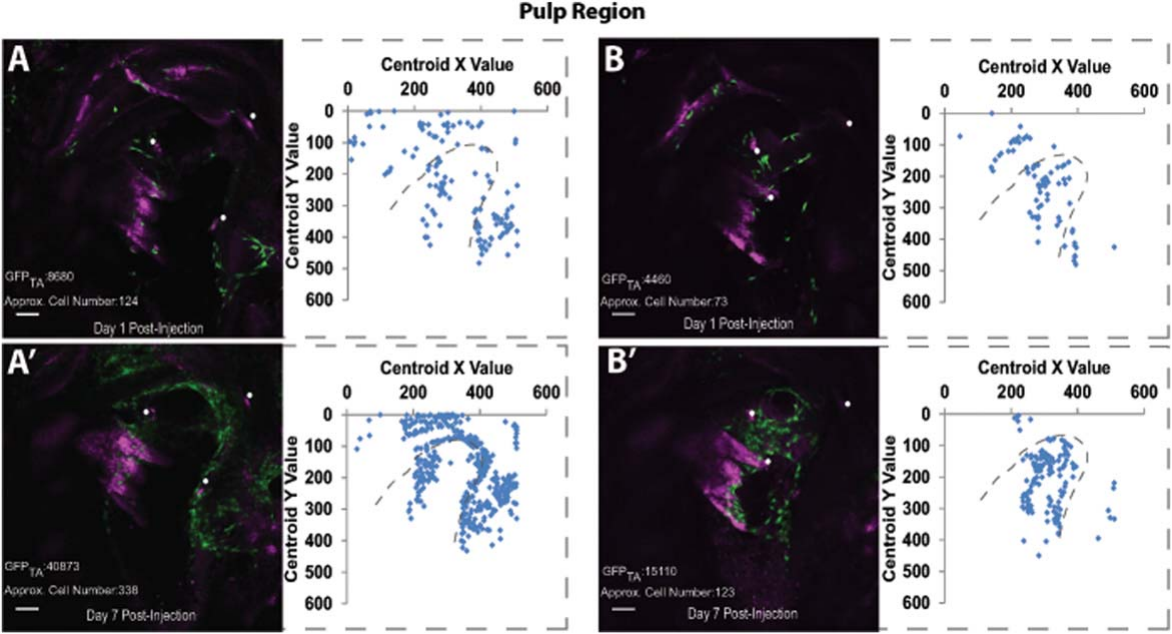
Representative confocal images from a mandible slice at days 1 and 7 post‐injection. **A**: Surface pulp region demonstrates cGFP‐DPPCs identification and location at day 1 post‐injection (tissue depth = 22 µm; GFP_TA_ = total area of GFP fluorescence in pixels). Magenta represents the reflected light channel and has been used to orientate the slice for image comparison, indicated by the white circles on each image. Approximate cell number is given and location of these cells has been plotted. The grey dashed outline is the approximate location of the dentine/pulp regions. **A'**: Cell location in the surface pulp region at day 7 post‐injection. **B**: cGFP‐DPPCs in sub‐surface pulp region at day 1 post injection (tissue depth = 15 µm). **B'**: cGFP‐DPPC expansion in sub‐surface pulp region at day 7 post‐injection. Scale bar = 100 µm. [Color figure can be viewed in the online issue, which is available at wileyonlinelibrary.com.]

The cGFP‐DPPCs were injected into the dental pulp of a rat tooth slice. After DAY 1 post‐injection, cGFP‐DPPCs were distributed in clusters across the surface pulpal region of the slice (Fig. 3A); by DAY 7 the cGFP‐DPPCs have expanded in the same region (Fig. 3A'). This is evidenced both by an increase in the total area of GFP (GFP_TA_), or total extracted cell number in the designated cell locations. From these simple analyses cell number expansion increased by >2.5‐fold (Figs. 3A and 3A'), and the total area of GFP signal (GFP_TA_) demonstrated a >4.5 fold increase in area. Similar clustered populations are seen on Day 1 in the sub‐surface pulpal region and by Day 7 the cells have expanded (Figs. 3B and 3B'). This is again confirmed by a cell number fold‐increase of 1.7 and a total GFP area that increase by approximately 3.5 fold between Day 1 and Day 7 (Figs. 3B and 3B'). In each case, reflecting the cell proliferation and the change in the underlying morphology of the cells. What is clear is that the distribution and location of the seeded cells is already determined by Day1, and that regional clonal expansion was detectable and quantifiable. The interplay between cell motility and proliferation would suggest that the latter is the predominant behavior between these two time points; at least from a global view.

**Figure 4:**
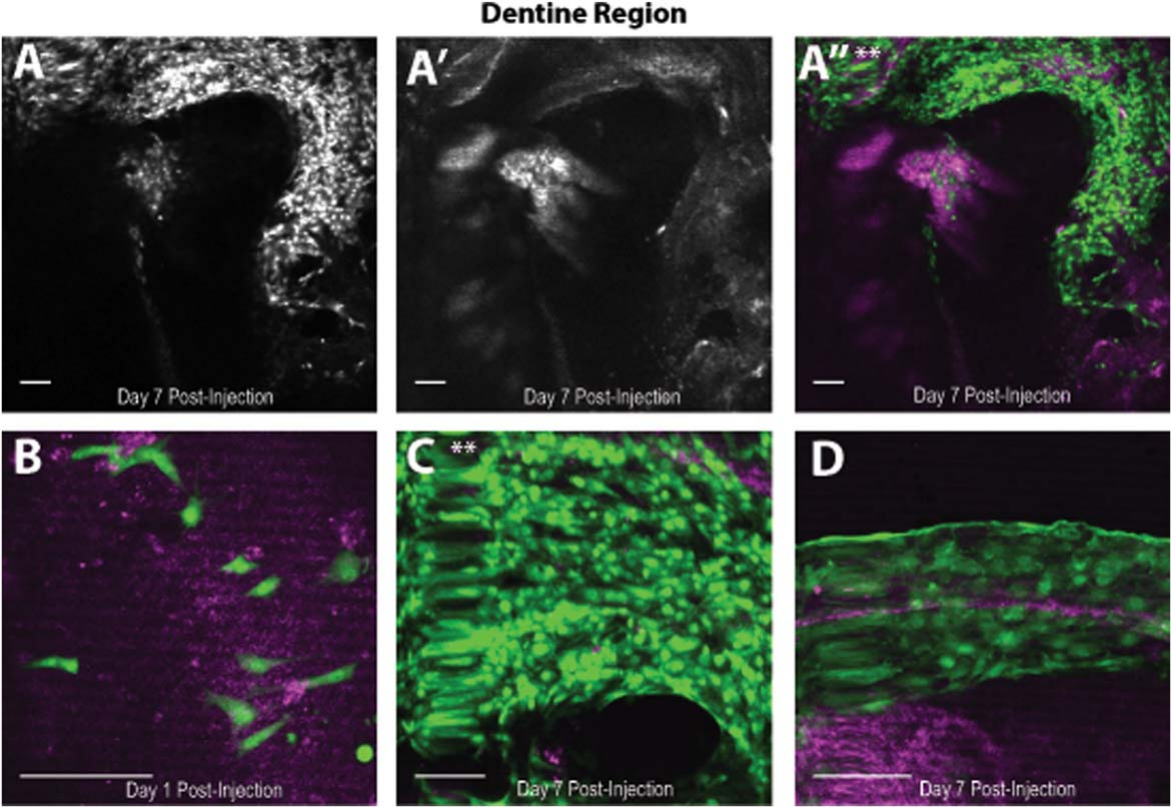
(**A**) cGFP‐DPPCs migration/expansion in the dentine region of the slice at day 7 post‐injection (tissue depth = 37 µm). **A'**: Reflected light. **A"**: Merged image. (**B**) Higher magnification image to show changes in morphology to and patterning day 7 post injection. ** indicates location of cells in A". (**C**) Higher magnification image to demonstrate morphology of cGFP‐DPPCs at day 1 post‐injection. (**D**) A different mandible slice focusing on the dentine surface demonstrating cGFP‐DPPC columnar morphology. Scale bar = 100 µm. [Color figure can be viewed in the online issue, which is available at wileyonlinelibrary.com.]

After 7 days of culture, on the upper surface of the tooth slice there were a large number of cGFP‐DPPCs that appeared to associate with the dentine surface of the mandible slice as evidenced by the reflection image (Figs. 4A,4A', and 4A"). The region is densely packed, along the dentine edge. A typical progression of proliferative expansion; cell packing and orientation, as well as drastic changes in cellular morphology were demonstrated as follows: Cells identified at DAY 1 post‐injection are few in number and have a spindle‐shaped cell body with outgrowth, and appeared to have attached to the tissues of the mandible slice as indicated by the reflection image (magenta) (Fig. 4B). By DAY 7, there was evidence of substantial cell alignment with cells taking on a columnar morphology at this surface (Figs. 4C and 4D), and including a highly dense region of cells alongside.

## Discussion

We are fundamentally interested in understanding how cells that grow in micro‐communities respond under tissue repair. This is not a trivial problem, and one that requires new tools and smarter “biomaterial” models. Due to large interplay of environmental factors that dictate mesenchymal progenitor cell behavior within the dentin‐pulp complex, advances in our understanding of dental pulp biology can only come with the development of more complex models that resemble the actual *in vivo* situation. It is to this end that we have described the development of a model that combines the use of an established *ex vivo* organ culture system, which allows cell behavior to be monitored and measured in the native 3D tissue environment. The current work relates to the reduction of excessive animal model systems for understanding bone repair by the creation of an organ‐like system, which could be used for the testing of compounds directly in human‐derived material. The requirement for such alternative translational *in vitro* systems allowing appropriate and reliable testing of new pharmaceuticals is of paramount importance 26. Others have assessed the osteogenic potential of human dental pulp stem cells on different polycaprolactone (PCL) scaffolds, determining their characteristics for promoting cell migration and differentiation 27. Here we describe a significant step toward being able to track dental‐pulp derived mesenchymal progenitor cells within their native extra cellular matrix environment in real‐time. This dynamic organ culture model system will prove to be useful in further exploring mesenchymal progenitor cell biology and for addressing questions that conventional 2D culture systems cannot, regarding the influence of pulp extracellular matrices and growth factors on maintaining “stem‐ness,” determining cell‐lineage fates and cell responses to tissue injury 28.

The cGFP‐DPPCs we have isolated and characterized to carry out this work will continue to provide a useful tool to further interrogate MSC biology as it pertains to clonal variation, control of differentiation, maintenance of pluripotency as well as behavior in complex 3D tissue environments. One of the difficulties in pursuing these lines of inquiry have been the lack of an ability to simply live‐image MSCs in order to track their lineage and relative location in complex culture environments such as whole tissues or tissue engineered scaffolds. These cells lend themselves to overcoming this barrier by producing a stable and easily accessible fluorescent readout that is sufficient to determine cell location, orientation and morphology. The methodology of injecting into the dental pulp area of the tooth and the subsequent mounting of the mandible slices into hydrogel proved effective from different perspectives for a longitudinal culture study. First, it provided a robust immobilization of the mandible slice in the imaging chamber, and second it provided a suitable optical interface (refractive index 1.365) between tooth biomaterial and coverslip, as well as a leveling surface for mounting the hard material as close as possible to the coverslip. Finally, the thermo‐reversible nature of this hydrogel permitted recovery of the slice ensuring the slices could be recovered and placed back in the conditioned (original) media.

Here we show the expansion of the injected cGFP‐DPPCs onto the cut surface of the dentine and the changes in their morphology from rounded to flattened and elongated. It has been well established that mineralized tissues, specifically both bone and dentine, contain a significant reservoir of sequestered bioactive factors 29, 30, 31, 32, 33, 34. Here it is possible that when presented with a “damaged” dentin surface, these factors were at work in recruiting the injected cGFP‐DPPCs to the site of the “injury.” Osteopontin is expressed by cells in a variety of tissues, including bone, dentin and hypertrophic cartilage; it is a key early marker of mineralized tissue matrix repair and has been postulated to play a role in initially attaching migrated DPPCs to a repair site 35, 36. Therefore as a potential robust differentiation biomarker, the expression profile in this mandible slice model requires further characterization. What is evident from these results is that, although the injected cells were of clonal origin, the cells have exhibited very different behaviors. Following injection, cells have expanded considerably over the 7 days in the cut dentine region whilst others remained in the pulpal soft tissue where expansion occurred to a lesser extent, which suggests that the cells are heterogeneous in nature. This is potentially due to differences in the differentiated state of the cells upon injection into the tooth slice: lineage‐committed phenotypes may migrate to the site of damage to initiate the repair process whilst more immature progenitor cells settle within a supportive niche environment, available for further recruitment 5. In the 3D mandibles the property of cellular expansion is clearly maintained, further experiments with single cell‐lineage tracking or barcoding would provide the opportunity for mapping clonal expansion in the native pulp and also to compare against the 2D model. There are also potential changes in morphology taking place over the 7 days. This is evidenced by the increase in the total GFP intensity/cell. This suggests that cells are potentially increasing in size or changing their morphology in response to their surroundings as the cells begin to interact with their matrix and other cells. Further investigation of changes to cell morphology is required to confirm this.

While cells were successfully injected into the dental pulp, control over the localized delivery of DPPCs in this model could be greatly improved. Due to the fluid dynamics of the injection media, injected cells had a tendency to disperse around the injection site rather than being delivered in a localized cluster. Use of an injectable cyto‐compatible material, such as a sheer thinning multi‐domain peptide scaffold as a carrier may allow for tighter plugs and more localized distribution of cells within specific regions of the dentin‐pulp complex 15, 37. Similarly, the model presented here could be used to examine the activity of other progenitor cells such as periodontal ligament or alveolar bone derived MSCs in their native 3D matrix environments, as these are present within the mandible slice organ 38. Given the interest in the use of progenitor cell based therapies to drive tissue regeneration, we propose that this model system could be used to gauge the response of intact tissues or biomaterials to the delivery of progenitors which are traceable/trackable in terms of their migration and differentiation once delivered 39, 40

## Supporting information

Supporting Information Figure 1.

Supporting Information Figure 2.

Supporting Information MIFlowCyt Item Checklist
