# Supplementary figures and images for: A 3D ex vivo mandible slice system for longitudinal culturing of transplanted dental pulp progenitor cells
Source: Cytometry A. 2015 May 11;87(10):921–8. doi: 10.1002/cyto.a.22680 (PMC4973699; doi:10.1002/cyto.a.22680)

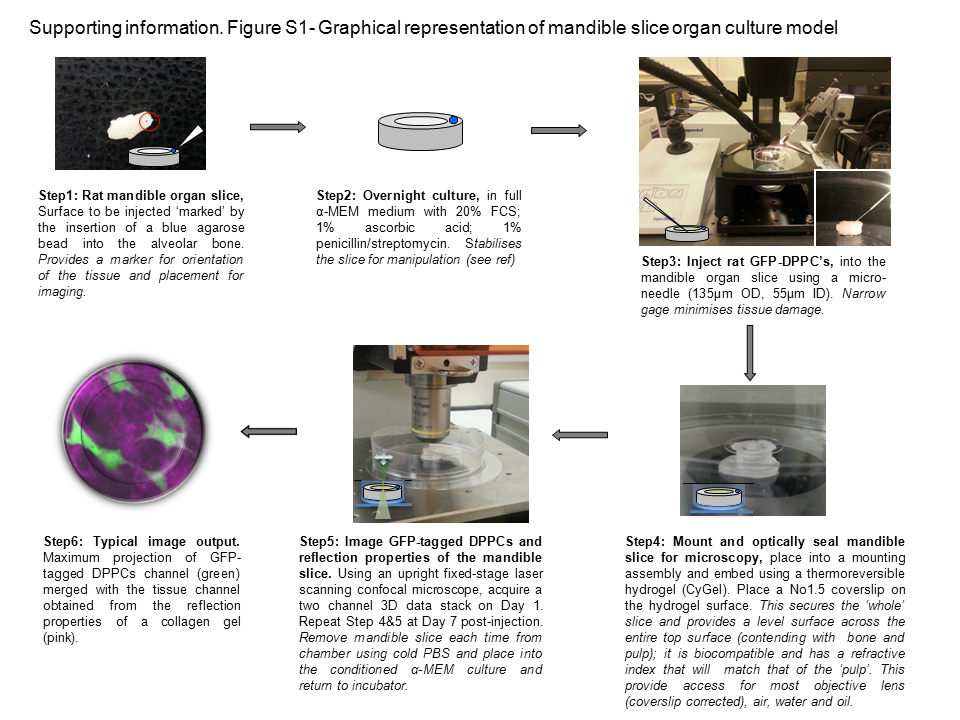

Supplement: Supplementary file 1 — Supporting Information Figure 1. [file CYTO-87-921-s001.tif]

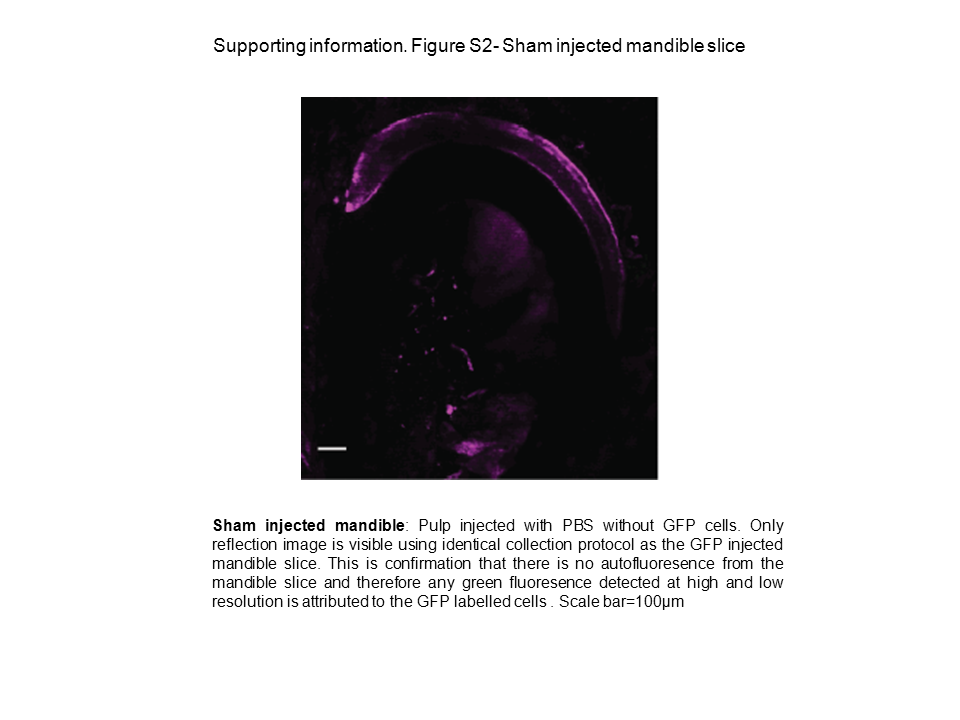

Supplement: Supplementary file 2 — Supporting Information Figure 2. [file CYTO-87-921-s002.tif]
