# Supplementary material for: A 3D ex vivo mandible slice system for longitudinal culturing of transplanted dental pulp progenitor cells
Source: Cytometry A. 2015 May 11;87(10):921–8. doi: 10.1002/cyto.a.22680 (PMC4973699; doi:10.1002/cyto.a.22680)
Supplement: Supplementary file 3 — Supporting Information MIFlowCyt Item Checklist [file CYTO-87-921-s003.doc]

**Cytometry Part A**

**Author Checklist: MIFlowCyt-Compliant Items**

| **Requirement** | **Please Include Requested Information** |
| --- | --- |
| 1.1. Purpose | The purpose of the flow cytometry was to identify what proportion of the Rat GFP DPPCs were expressing GFP prior to transplantation into the mandible slice. |
| 1.2. Keywords | GFP, dental pulp progenitors |
| 1.3. Experiment variables | N/A |
| 1.4. Organization name and address | Cardiff University, Institute of Cancer and Genetics, Tenovus Building, Heath Park Campus, Cardiff, CF14 4XN |
| 1.5. Primary contact name and email address | Dr Rachel Howard-Jones HowardjonesRA1@cf.ac.uk |
| 1.6. Date or time period of experiment | 22/02/2012 |
| 1.7. Conclusions | GFP was highly expressed in the rat DPPCs prior to transplantation |
| 1.8. Quality control measures | GFP expression was assessed in cells under normal culture conditions. |
| 2.1.1.1. (2.1.2.1., 2.1.3.1.) Sample description | GFP DPPCs isolated from ‘green’ rats. For more information see material and methods section |
| 2.1.1.2. Biological sample source description | DPPCs isolated from rat mandible and selected based on adherence to fibronectin |
| 2.1.1.3. Biological sample source organism description | Rat |
| 2.1.2.2. Environmental sample location | N/A |
| 2.3. Sample treatment description | Prior to flow cytometry analysis cells were cultured under normal growth conditions. See materials and methods for details. |
| 2.4. Fluorescence reagent(s) description | N/A |
| 3.1. Instrument manufacturer | Becton Dickinson |
| 3.2. Instrument model | FACSCalibur |
| 3.3. Instrument configuration and settings | 488nm 530/30nm BP |
| 4.1. List-mode data files | The repository identifier:  <http://flowrepository.org/id/FR-FCM-ZZJ6>. |
| 4.2. Compensation description | Flow cytometry assay was single colour so no compensation was required. |
| 4.3. Data transformation details | Data was analysed offline using FlowJo |
| 4.4.1. Gate description/statistics/boundaries | 10000 events were collected for analysis. Forward and side scatter was used for gating to eliminate cellular debris and cell doublets from analysis. Further gating or two distinct cell populations was applied to establish proportion of DPPC expressing GFP. |

**Notes**

Feel free to use more space than allocated.

You can embed graphics/figures in this document, if needed.

Please make sure to save the document in Microsoft Word version 2003 or older, before uploading to ScholarOne Manuscripts. When uploading this checklist to ScholarOne Manuscripts, please choose the “Supplementary Material for Review” category.

Please note that if your paper is accepted, the checklist will be published as an Online Supporting Information.

For any questions, please contact the Cytometry Part A editorial office at [Cytometrya@wiley.com](mailto:Cytometrya@wiley.com).
